# A face of one’s own: The role of an online personae in a digital age and the right to control one’s own online personae in the presence of digital hacking

**DOI:** 10.1007/s12525-024-00713-3

**Published:** 2024-04-30

**Authors:** Eric K. Clemons, Andrej Savin, Maximilian Schreieck, Stina Teilmann-Lock, Jan Trzaskowski, Ravi Waran

**Affiliations:** 1 The Wharton School, University of Pennsylvania, 3730 Walnut Street, 572 Jon M. Huntsman Hall, Philadelphia, PA 19104 USA; 2 Department of Business Humanities and Law, Copenhagen Business School, Solbjerg Plads 3, 2000 Frederiksberg, Denmark; 3 Department of Information Systems, Production and Logistics Management, University of Innsbruck, Universitätsstraße 15, 6020 Innsbruck, Austria; 4 Clearwater Paper Corporation, 601 W. Riverside, Suite 1100, Spokane, WA 99201 USA

**Keywords:** Online personae and identity, Online personae and identity hacking, Disinformation, Algorithmic control of disinformation, D60, K24, L15, L50, L86, C63

## Abstract

In the post-Covid world, our online personae have become increasingly essential mechanisms for presenting ourselves to the world. Simultaneously, new techniques for hacking online personae have become more widely available, easier to use, and more convincing. This combination, of greater reliance on online personae and easier malicious hacking, has created serious societal problems. Techniques for training users to detect false content have proved ineffective. Unfortunately, legal remedies for dealing with hacked personae have also been inadequate. Consequently, the only remaining alternative is to limit the posting of false content. In this discussion paper, we provide an overview of online personae hacking. As potential remedies, we propose to redesign search engine and social media algorithms allowing platforms to detect and restrict harmful false content and a new fundamental right for the EU Charter that would provide legal justification for platforms to protect online reputations. For those platforms that might choose not to protect online reputations, this new right would require that they do so.

## Motivation

The importance of our online personae is evident from the care we give it, including ego surfing to see how we look to others and adding photographs of our accomplishments (Barbour et al., 2017), even when these posts can create significant personal risk (Shulman, 2022). This concern for our online personae is reasonable, given the use of these personae for tasks as diverse as preparing for a business meeting, evaluating a job candidate, or dating. Experienced online users use self-promotional search engine optimization to ensure they are found when they want to be. Teens and members of Gen-Z, the leading wave of digital natives, are known to curate their online personae carefully (Barbour et al., 2017; Katz et al., 2022).

Recently, techniques for hacking an individual’s online personae have increased enormously. Traditional forms of deliberate persona hacking have existed for decades, perhaps most famously starting with Richard Nixon’s telephone-based hack of Helen Gahagan Douglas in the senate campaign of 1950, falsely “informing” tens of thousands of California voters that Douglas was a card-carrying member of the Communist Party. Persona hacking has gained power, speed, and impact since then, aided by our reliance on online personae and the ease of manipulating these online personae (e.g., van Huijstee et al., 2021). Persona hacking is further accelerated by AI techniques for the generation of fake narratives through text generation programs like chatbots (Mansfield-Devine, 2023; Weil, 2023) and sophisticated graphic tools for deepfakes (Hancock & Bailenson, 2021; Metz, 2023).

Legal scholars assure us that most active and intentional defamation forms are illegal. However, our research shows that this offers, at best, limited protection from the harm created by online personae hacking (Revilla, 2017). The harm from persona hacking is immediate, as with Nixon’s disinformation campaign, hours before the election. Refutation and removal of content is slow and uncertain (Claypoole & Payton, 2016) and cannot always be accomplished before irreparable harm is done. Restitution is even slower and even more uncertain. Complicating the problem of restitution, it is difficult to establish the relationship between a harmful outcome and any single fake narrative among many. Even the EU’s proposal for the AI Liability Directive, which explicitly bans subliminal manipulation through AI-generated deepfakes, offers little protection from sophisticated deepfakes other than declaring that the material is illegal—what, exactly, can be done to eliminate the harm created by a convincing video of a presidential candidate weeping in front of a judge, pleading his innocence, and begging to be released from custody? Once seen, its impact lingers, either loathing for the object of the video or sympathy and support.

Humans have proven remarkably resistant to attempts to wean them from fake news, either by debunking and labeling stories as false or by pre-bunking, training them to recognize and reject fake narratives (A. Kamenetz, 2017; Kim & Dennis, 2019; Kim et al., 2019a, 2019b; Roozenbeek & Linden, 2019; van der Linden et al., 2021). This will only get harder as deepfakes become more convincing, and a flood of chatbot-generated false content alters the balance between truth and fiction on the net. If correction is slow and uncertain, and if training users to detect disinformation and persona hacking is difficult or even impossible, the solution must involve debunking and pre-bunking the algorithms serving us content. We cannot stop the creation of false narratives and deepfakes. But we can ensure that fake content is never the first thing returned in a search and is never the first item in our automated news feeds.

In this discussion paper, we draw on previous work on online personae and online defamation (Barbour et al., 2017; Claypoole & Payton, 2016; De Kerckhove & Miranda De Almeida, 2013) and argue that a comprehensive overview of types of hacks of online personae and the state of legal protection for these hacks is missing. Such an overview, however, is instrumental to discussing how we can achieve better protection of online personae. Therefore, we examine a large set of abuses until we believe we have a “spanning set” of significant forms of persona hacking available now or in the immediate future. We then develop a minimal classification that allows us to characterize every element in the set. We evaluate the legal history associated with each, including whether or not litigation was deemed possible, whether or not litigation was initiated and concluded in favor of the victim of persona hacking, and, ultimately, whether or not the victim was adequately compensated and adequate restitution was available. We examine the impact of current and future technologies on the prevalence and virulence of this form of persona hacking. And where we conclude that the problem will become more severe over time and that restitution is not and indeed cannot be sufficient to undo harm, we suggest that new techniques are essential to prevent that harm.

We next introduce the concept of an online personae and the significance of online personae hacking. The next section of the paper provides a literature review. First, we review the significance of public personae in literature and philosophy; then, we review literature on online personae hacking. Next, we review forms of relief that appear to be available to an individual who has suffered online personae hacking and how these protections are currently inadequate. We then present our classification and examination of various forms of online personae hacking. We suggest that the best way to protect online personae in the EU is the creation of a new fundamental right to an unharmed online persona, and we propose an algorithmic approach that platforms could be required to implement to ensure this new fundamental right. The final section presents our conclusions, including suggestions for future research.

## Background and literature review

### Definition of the term online personae

An online personae is “a part of the individual identity that has been extended into the online sphere” (De Kerckhove & Miranda De Almeida, 2013, p. 277) through the various data we post, transactions we engage in, and traces we leave online (Clarke, 1994, 2014). In a post-Covid age, our online personae defines us in public life, employment, university admissions, the decision to enter into commercial interactions, and a wide range of other professional and social settings. The online personae is a first test or screening procedure, which must be deemed acceptable before more serious interactions can commence. These online personae are different from commercial profiles, maintained by companies based on our transaction histories, and often invisible to us and impossible for us to control. For example, platform providers like Amazon build commercial profiles of their customers which can be used to serve customers with targeted ads and recommendations. In contrast, our online personae is visible, notably on search engines and social media networks, and much of what we do online is designed partly to curate our online personae.

If having an acceptable online personae is the first hurdle an individual must pass, then damaging or destroying an individual’s online personae can create inestimable damage not easily imagined previously. If online personae were not previously of vital importance, and since online personae hacking has not previously been a serious problem, there is no reason to expect that there is already a robust body of law designed to limit this form of abuse.

Hostile online personae hacking is separate from persona enhancement performed by the individual for personal gain: these activities can range from adding enhanced and more attractive digital images (Bell, 2019) to the fabrication of an entirely false history, education, religion, and national origin in a political candidate such as the former Congressman George Santos who was expelled from the House of Representatives after the allegations came to light (U.S. Attorney’s Office, 2023; Gold & Ashford, 2023). These are not problems of the same type as hostile hacking of an individual’s reputation, but they demonstrate the importance we now place on our online personae. As discussed throughout this paper, the decision about what individuals can and cannot say about themselves should be subject to more scrutiny. But that is separate from the issues associated with hostile persona hacking by others. Furthermore, persona hacking is different from the increasing problem of online bullying, increased depression, and increased incidence of self-harm as a result of abusive behavior on online social networks (Sadagheyani & Tatari, 2021). While we do not specifically address online bullying, we note it is a significant social problem.

We note the differences among one’s legal identity, online personae, and online roles in passing. One’s *legal identity* is usually fixed and immutable unless you are entered into something like a witness protection program and issued a newly fabricated identity. One’s *online personae* is how you are seen online (Moore et al., 2017). Some of this is subject to individual control, while other aspects of your online personae are less easily controlled. It is not necessary to have only a single online personae because we play different roles in our lives. As a result, it is more challenging to establish rules determining what changes to an individual’s online personae should and should not be permitted over time.

Our proposed regulatory responses to the problem of hacking an individual’s online personae are twofold. The first step is to create a new fundamental right in the EU, protecting one’s online personae from hostile attacks. The second step is to require online platforms to implement algorithms that protect online personae from attack.

The right to maintain an online personae free from attack is far more complex than the legal “right to be forgotten,” more accurately termed the “right to be forgiven.” The “right to be forgotten” gives individuals the right to influence what can and cannot be returned through an online search, though not the right to determine what is present online, and even this limited right is subject to continued analysis and dispute (Ausloos, 2020; Lambert, 2019; Martínez & Mecinas, 2018; Werro, 2020). As discussed throughout this paper, the decision about what can and cannot be posted about individuals by third parties remains far more complex and is far from resolved by existing legal doctrines.

### Roles and importance of a public persona throughout history

As noted above, in the history of the importance of the public persona, the Aristotelian *zoon politikon* may be perceived as a variant of it. In particular, the public persona is the single agent only to be realized in the active life of the city and the business of politics (Dossa, 1989; Owens, 1988). Since antiquity, the public persona has been seen in extension or tandem and sometimes in opposition to an inner self.

In the nineteenth century, the American psychologist and philosopher William James introduced the distinction between the “spiritual self” and the “social self” (James, 1890). Today, this distinction is often referred to as one between the “private” and the “public” selves (Lamphere & Leary, 1990). Along these lines, the twentieth-century American social theorist George Herbert Mead introduced the notion of the social self, emphasizing symbolic interaction over time with other people in forming the social self (Mead, 1982).

Nietzschean and Freudian idealizations of psychological authenticity (Stolz, 2020) formed the basis for the Swiss psychiatrist Carl Jung’s influential concept of the “persona,” through which Jung critically addressed the potential for public “false” or fabricated selves (Jung, 1953). Notably, Jung describes a “complicated system of relations between individual consciousness and society,” where the persona amounts to “a kind of mask designed to ‘impress and conceal’ and to meet societal demand” (Fawkes, 2015).

The tradition from the North American sociologist Erving Goffman has been privileging the study of “face-work,” defining public “face-to-face” interaction as what people do in the process of claiming “positive social value”—including reputational value—in social contexts. In Goffman’s theoretical framework, a person’s “face”—and public persona and reputation—constantly evolves depending on societal rules and values (Bullingham & Vasconcelos, 2013; Goffman, 1956; Raffel, 2013).

A significant theorist of the ethical aspects of the “face” and face-to-face interaction is the philosopher Emmanuel Lévinas, who designates “the face” as a fundamental point of reference in the ethics of interaction. Lévinas employs the notion of the “alterity” of the Other, as signified by the “face.” In other words, the face is something that one acknowledges before using reason to form judgments or beliefs, and utilizing the face creates an ethical relation via a mutual presentation of “self by self” (Levinas, 1961). It is “this Other” that “speaks to me, implores or commands me” (Bergo, 2019). Crucially, it is also in response to the “Other” that moral responsibility is constructed (Casey, 2006). Thus, the public persona has long been important for human interactions—and will continue to be important in the digital era.

### The emerging problem of online personae hacking

Today, technologies that allow for hacking public online personae are available to anyone with a computer and average technical skills. Notably, aspects of public personae represented online, including faces, names, voices, bodies, and movements, can be selectively amplified, omitted, juxtaposed, and recontextualized. One effective online technique includes search engine manipulation and distortion, which alters what users find during a search. Recent statistics provided by Google show that defamation and impersonation are the most important reasons for requests for content removal in the US and India (Google, 2022, 2023). Another is the platforms’ creation of echo chambers and satisfaction bubbles in social media, feeding users content that will produce strong emotional responses, regardless of accuracy, euphemistically called “increasing the quality of the user experience.” With new techniques, for example, deepfakes, over-dubbing, voice cloning, web browser manipulation, internet bots, natural language processing, generative pre-trained transformers, or manipulation of social media profiles and Wikipedia articles, any online personae can be credibly recreated in an artificial context that appears to depict someone, although it is not (Chesney & Citron, 2019; Harris, 2019; Heugas, 2021; Kietzmann et al., 2020; O'Connell, 2020).

Various forms of technology make fakes, hacking, and long-term coordinated disinformation campaigns easier. The technology behind the original Avatar movie was expensive and beyond the reach of most home hackers; today, anyone with sufficient interest and motivation can generate convincing fake videos at home (Finger, 2022). It once took a staff of writers to develop a coordinated disinformation campaign, but chatbots and predictive text generation systems can enable a single individual to generate a volume of disinformation that once would have required a staff (Bond, 2021). These are not the principal reasons that Hinton, the “godfather of AI,” considers AI a greater danger than climate change (Metz, 2023), but they do need to be considered on any list of potential threats from AI.

Hacking of online personae that involves damage to political interactions opens up a host of societal vulnerabilities: it is not necessary for a hostile foreign power to convince the political body of any individual falsehood merely to convince the electorate that *no one* and *nothing* is to be trusted (Baghramian, 2020). Selective presentations of half-truths—for example, publicizing an opponent’s erroneous arrest while omitting the fact that charges were immediately dropped—represent a problem to individuals and companies, as do careful construction and targeting of online misrepresentations or false histories that would be effective for influencing consumers or voters (Langa, 2021; Spivak, 2019; Susser et al., 2019). As has been documented in studies by the EU Parliament, there is an upward trend in targeted personal hacking and disinformation attacks (van Huijstee et al., 2021). An integrated survey by the UK House of Commons (2018) presented evidence of systematic disinformation campaigns to interfere with elections, and individual accounts are available in the press (Woodcock, 2020). The reputational hacking of public persona, including politicians, frequently goes beyond one-off individual events and often involves long-lasting, ongoing coordinated campaigns, sometimes by domestic groups with extreme views and increasingly by foreign fake news and foreign discord-exporting factories.

While previous literature acknowledges the increasing prevalence of persona hacking, a comprehensive taxonomy that considers the technique of the hack, its impact, and potential protection against it is lacking. Previous work either comments on specific techniques such as deepfakes (e.g., van Huijstee et al., 2021) or takes on a broader view without aiming to provide a systematic overview of hacking techniques (e.g., Claypoole & Payton, 2016).

## Classification of types of hacks against online personae

We next present our taxonomy of the nature of hacks against online personae. This classification is helpful because different forms of attacks will exploit different limitations in current legal protections and may need regulators to create different forms of protection to restrict their occurrence. The taxonomy is both inductive and deductive. On the one hand, we looked at how an individual’s persona could be hacked and then made a mental list of the possible hacks. We conducted extensive online searches for hacks and online defamation examples to make that list as complete as possible. Then, we compared that list with literature related to hacks of online personae (e.g., Alavi, 2018; Albahar & Almalki, 2019; Claypoole & Payton, 2016; Henderson et al., 2023; van Huijstee et al., 2021). We started by looking at whether the hack was text-based or more complex. For text-based hacks, we began by exploring the role of lying and expanded from there. With this taxonomy as part of a discussion paper, we aim to provide a starting point for future research to validate, refine, and update the taxonomy and analyze potential remedies.

We will elaborate on text-based hacks in the first subsection (see also Table 1), while we address more complex audio- and audio–video-based hacks in the second sub-section (see also Table 2). Both tables present the *name of the technique*, a *brief description*, and a review of the *change in status* of the technique, which is the extent to which technology has made the technique more significant. The tables also include one or more *lead examples* where the technique is used and the current *status of protection* available to victims of each form of online personae hacking. Table 2 is addressed in the following section.

### Text-based hacking

**Table 1:** Classification of attacks on an online personae based on the nature of the technology and the nature of the form of the attack (text-based attacks)

Name of technique	Description of technique	Change in status	Lead examples	Status of protection
Flooding the zone	Take a single true incident that is embarrassing to the individual and ensure through search engine optimization that accounts of this incident dominate search	Did not exist before search engines and the increasing importance and accessibility of online personae	Ben Edelman at Harvard; Google search results were biased to make him look ridiculous and a disgrace to Harvard	Appears to be legal because Google has established its right to return whatever search order it wants as a legally protected editorial opinion and because it is not writing any of the material it reports; likewise, social media platforms have not been held responsible for the accuracy of stories they show users
Replacement and Google bombing	Create an alternative and highly damaging magnet for users’ attention, combined with search engine optimization	Did not exist before search engines and the increasing importance and accessibility of online personae	George Bush merchandise directed to “Dumb Motherf*cker”; “More Evil than Satan” directed to Microsoft home page	Individuals may enjoy some protection if the campaign involves traditional media and malicious intent can be established; can also be treated as misleading business practice or as protection of reputation, but neither seems effective
High-impact lying	Add a compelling but false narrative to a true persona, combined with careful fake news dissemination targeting	Getting easier because we rely more and more on online personae, so there are more ways to hack	Sandy Hook Infowars, Smear campaign against commodity trader Hazim Nada	Clearly illegal defamation, but that does not appear to have provided necessary protection in many cases; the successful suit against Sandy Hook Infowars is a rare and highly visible example of success
Alternative facts and stochastic truth	Flooding the web with numerous false stories, now rapidly created by chatbots and predictive text generation programs	Did not exist before the web and getting easier because of chatbots and robotic text generation programs; newly significant because it is newly easy	Ongoing campaigns against Hillary Clinton; conspiracy theories related to George Soros, ObamaGate	Clearly illegal defamation, but that does not appear to have provided the necessary protection

*Flooding the zone* is a form of search engine manipulation in which the search results returned first are most damaging to the individual that is the object of the search rather than those that are most relevant and most important to the searcher. Ideally, in this technique, the search results in true stories that have been published and were not written by the parties responsible for the attack on the online personae since it is impossible to establish libel when this is true. This form of attack did not exist before online personae and online search. It is extremely difficult to prove since search algorithm details are always closely guarded secrets. Occurrences of this form of persona hacking may be difficult even to detect for the same reason: it is not always apparent that the order of search results has been manipulated to harm an online personae. And it is probably impossible to prosecute; the stories returned by search in this form of attack are all true, and none were written by the creators of the attack, so legal actions claiming slander or libel would be impossible.

We are aware of only one example of this form of hack against an online personae, perhaps because it occurs rarely or is challenging to detect. The example involved Ben Edelman, an Associate Professor at Harvard who was a frequent and influential outspoken critic of online platform companies. He got involved in a dispute with a local Chinese restaurant over a four-dollar discrepancy in an online takeout order. The Chinese restaurant took the story to a local newspaper, and it rapidly achieved national prominence, with bloggers competing to see which could be more vitriolic; see, for example, Berman (2014). Even national media got into the act, with New York Magazine opening its story with “Internet, meet your new laughingstock” (Bankoff, 2014). The attacks on the professor were positioned at the top of search results, so they soon overwhelmed and obscured references to the professor’s research and academic websites. The embarrassment this caused to the professor’s colleagues and home institution might have contributed to the decision to deny him tenure. While Professor Edelman is not suing Google for the way they handled the ordering of search results, he has recently filed suit against Harvard for the way these search results were interpreted during his tenure review (Burns, 2023). The legacy of this incident continues to plague Edelman; see, for example Bryne (2023).

*Replacement and Google bombing* are sophisticated forms of search engine manipulation. Adam Mathes originally coined the term (Wikipedia, 2023a). Google bombing occurs when a popular search string is redirected to an alternative site, obviously associated with an individual or organization’s online personae, when the site is offensive and damaging to the persona. The best-known examples involved having searches for George Bush merchandise directed users to a site “Dumb MotherF*cker” (Wikipedia, 2023b) and a search for “More evil than Satan” directed users to Microsoft’s corporate home page (Brady, 2004; Hamilton, 2013).

Less scalable forms of this attack have existed before; gossip could replace truth, as amusingly illustrated in Chekhov’s “The Kiss” (Chekhov, 2000). But as online personae and online search become more important, the opportunities to manipulate an individual’s persona by planting alternative personae on the web and redirecting users to them increase. Legal remedies would appear to be limited. The injured party can demand that search algorithms be corrected, but at present, Google has successfully defended its search result orderings as protected editorial opinions, defended by the First Amendment (Stern, 2014).

*High-impact lying* occurs when a false story is created and passionately defended online. One of the most famous examples is Alex Jones of Infowars repeated claims that the massacre at Sandy Hook Elementary School never occurred and accusing the parents of the murdered children of conspiracy (Wikipedia, 2023c). The massive judgments against Infowars and Alex Jones (Williamson, 2021) totaled nearly $1.5 billion in Connecticut (Darcy & Maghe, 2022), in addition to a smaller judgment in Texas (Vertuno, 2022). This outcome can be expected to limit the profit-seeking big lies of individuals like Jones.

In a recent example from the business world, the American commodity trader Hazim Nada was the victim of a smear campaign that forced his shipping and trading company into bankruptcy. Numerous reports linked the company to terrorist activities, destroying its reputation and credit (Kirkpatrick, 2023). Nada filed a lawsuit against several parties in January 2024 at a Washington DC court, including the United Arab Emirates and its national oil company, accusing them of a defamation campaign (Farchy, 2024). The outcome of the litigation is still unknown. However, the lawsuit is only possible because documents were leaked from a private intelligence firm that provided details on the smear campaign. Without such evidence, making a case for defamation would be difficult.

*Alternative facts and stochastic truth* occur when the volume of deliberately false information relevant to a search, the result of an ongoing disinformation campaign, begins to match the volume of true information relevant to the same search; this overwhelms the search engine’s ability to distinguish fact from fabrication. This form of hack is distinct from high-impact lying, which relies on a small number of compelling lies. In contrast, stochastic truth relies on the sheer volume of falsehoods to obscure the truth.

Lying is not new, but persistent online disinformation campaigns can be damaging in ways that word-of-mouth lying might not have been, especially when the volumes become sufficient to allow the lies to appear comingled with mainstream information in search results. Examples include the decades-long attack on Hillary Clinton (Goldberg, 2016). Former president Trump’s steam of false claims about his predecessor Obama rose to the level of stochastic truth (John, 2020), attempting to overwhelm reality by sheer numbers and repetitions. Search engines can be deceived, and predictive text generation algorithms and chatbots threaten to overwhelm the web with vast numbers of false stories created effortlessly on demand (Klepper, 2023). An example from the business world is George Soros, who has become a target of numerous conspiracy theories in right-wing media (Plenta, 2020).

Individuals are protected because deliberately false attacks are covered by antidefamation laws, but this protection is limited. As noted above, prosecution is time-consuming and uncertain, while the harm is demonstrable and immediate. And as AI-generated campaigns become more prevalent and extensive, individuals’ efforts will become less and less effective. At present, search engines cannot be compelled to adjust their algorithms. And at present, online social networks have been unwilling to cooperate since the most inflammatory articles generate the most engaged users and create a significant portion of the platform’s revenues (Vogelstein, 2018).

### Audio- and audio–video-based hacking

**Table 2:** Classification of attacks on an online personae, based on the nature of the technology and the nature of the form of the attack (audio- and audio–video-based attacks)

Name of technique	Description of technique	Change in status	Lead examples	Status of protection
Flooding the zone with chance but true juxtaposition	Giving artificial prominence to true photographic images of chance encounters or encounters that were not known to be significant at the time	Getting easier because we rely more and more on online personae, so there are more ways to hack	Jeffrey Epstein and connections to Harvard, MIT, and Bill Gates	Appears to be legal because connections were real, and disclosure forced admissions of responsibility and change of policy at Harvard and MIT
Isolation and omission	Involves removing some of the context to create a false impression of the context	Getting easier because we rely more and more on online personae, so there are more ways to hack, and because editing technology has improved	Editing an actual video to make Representative Ilhan Omar appear to think 9/11 was not significant	Should be protected as a form of defamation, but these protections have not been effective
False juxtaposition and splicing or misrepresentation	Similar to isolation and omission, but this involves creating artificial context; combining images to create a composite that never existed in reality	Getting easier because we rely more and more on online personae, so there are more ways to hack, and because editing technology has improved	Edited video of Belarusian missile training to create the impression that democratic representative is indifferent to rocket attacks on Israel. Pictures of Alphabet CEO Sundar Pichai taken out of context and combined with fake quotes	Should be protected as a form of defamation, but these protections have not been effective
Low-tech fake	False audio over true video, manipulated audio over true video	Getting easier because we rely more and more on online personae, so there are more ways to hack, and because editing technology has improved	Video of Nancy Pelosi is slowed down (and the speech altered to still sound like her original voice) and distributed widely	Should be protected as a form of defamation, but these protections have not been effective
Deepfake	Complete fake audio video, superior Al-created lipped synched false audio over true video	Getting easier because we rely more and more on online personae, so there are more ways to hack, and because editing technology has improved	Deepfake video of Mark Zuckerberg revealing the “truth” about privacy on Facebook; deepfake video of Ukrainian president Zelensky surrendering to Russia	Should be protected as a form of defamation and by the protection of the rights to one’s own image; alternatively, the action may be protected as a political parody; current protections for reputation do not appear effective

Table 2 presents the results of our analysis and continues our presentation of our taxonomy of the range of potential hacks of online personae. Table 2 addresses more complex audio- and audio–video-based hacks than in the previous sub-section.

*Flooding the zone with chance but true juxtaposition* refers to giving artificial prominence to true photographic images or video material. The result is reputational damage to the individual under attack. Flooding the zone attacks are getting easier and more impactful because we rely more and more on online personae, providing more targets and material for such attacks. Examples include pictures that show the connections of Jeffrey Epstein—a convicted sex offender who committed suicide in prison—to Harvard (Greenberg, 2019), MIT (Gilbert, 2019), and Bill Gates (Flitter & Stewart, 2021). Another example is the “Biden Blunders,” which refers to videos that show gaffes by Joe Biden and are circulated by right-wing media such as Fox News to harm Biden’s credibility (Gillespie & Cina, 2023).

This type of attack appears to be legal because the photos and videos show true material. Therefore, protection against these attacks is difficult. For example, the disclosure of Epstein’s connections to Harvard and MIT led to admissions of responsibility and policy changes at the two institutions (Bradt, 2020; Fox, 2021).

The *isolation and omission* attack involves removing some of the context to create a false impression of the context. Again, the attacks can result in reputational damage to individuals, and, again, they have become easier as we rely more on online personae. In addition, continuous improvements in editing technologies have made it easier to manipulate photo and video material. An example is a video snippet that shows Rep. Ilhan Omar apparently downplaying the 9/11 terror attacks. But if one considers the whole clip, this is clearly not what she is saying (Washington Post, 2019a). Similarly, Republicans shared a video snippet in which Biden stated that the Democratic Party has set up the largest “voter fraud organization”; however, he misspoke, and the whole speech is about fighting voter fraud (Reuters, 2020). While this type of attack could be interpreted as defamation, the protections have not been effective.

*False juxtaposition and splicing*, *or misrepresentation*, refers to an attack that combines photo and video material that never existed in reality or puts existing material in a false context to create a new, damaging narrative. This type of attack has also benefited from the improvements in editing technologies. An example includes video material that supposedly showed missiles being fired from Palestinian territory on Israel while, in fact, showing military training of the Belarusian army. Republican activists used the video to try to show that Representatives of the Democratic Party are indifferent to rocket attacks on Israel (Washington Post, 2019a).

In an example from the business world, pictures of Alphabet and Google CEO Sundar Pichai have been used to spread false narratives, particularly in India. One post falsely claimed that he was casting his vote in India, using an older picture depicting a visit of Pichai to his alma mater in India (Stalin & Roy, 2019). Another widely shared picture combined a portrait of Pichai with a fake quote about him getting involved in the discussions about a beef ban in India (TNM, 2017).

The *low-tech fake* attack refers to an attack that uses fake material that can be created easily without relying on sophisticated video editing or artificial intelligence. The most basic forms of this attack include putting false audio on a true video or manipulating the audio of a true video. For example, a video with altered audio of Nancy Pelosi has been circulated by Republicans. Pelosi’s speech is slowed and distorted, and the video’s captions typically questioned her fitness or state of mind (Washington Post, 2019b). Similarly, the White House under the Trump administration apparently published a slightly edited video of CNN reporter Jim Acosta pushing away the arm of a White House intern to justify that they revoked Acosta’s access pass. Only the movement of Acosta was sped up to make it appear more aggressive (Harwell, 2018). This type of attack should be protected as a form of defamation and by the rights to one’s own image, but these protections have not been effective.

The *deepfake* attack goes beyond the low-tech fake by using sophisticated technologies to create completely fake audio and video material or manipulate videos with “visual dubbing,” i.e., synching the lip movements of any speaker to a given audio. While visual dubbing can be helpful, for example, to provide a better experience for translated movies (H. Kim et al., 2019a, 2019b), it can also be used to create deepfakes (Westerlund, 2019). Deepfakes “leverage powerful techniques from machine learning and artificial intelligence to manipulate or generate visual and audio content with a high potential to deceive” (Kietzmann et al., 2020).

For example, a deepfake video of Meta-CEO Mark Zuckerberg showed him saying that Facebook manipulates its users (Posters, 2019). The caption of the Instagram post made it clear that it was a deepfake video and part of an art project; nevertheless, the video also circulated without the caption. In another example, a deepfake video of Ukrainian President Zelensky surrendering to Russia in the first weeks of the war in Ukraine was circulated on social media (Miller, 2022). The video was heavily doctored, with Zelensky’s head placed on a body and lip movements synced to the audio. However, it was easy to spot that the video was fake, most likely limiting its impact. Again, this type of attack should be protected as a form of defamation and by the rights to one’s own image, but these protections have not been effective.

## Recommendations

### Enhanced legal protection

Perhaps the best way to achieve enhanced legal protection in the EU would be as an extension to the Charter of Fundamental Rights of the European Union, which combines and states in one legally binding document the most critical personal freedoms and rights enjoyed by citizens of the EU. This right to an unharmed online personae may also be protected by some of the amendments in the United States Bill of Rights, although after the recent Supreme Court decision reversing Roe v. Wade, the role of privacy as a guaranteed constitutional right may now be uncertain (Gerstein & Ward, 2022). The problem is not that the EU or the US lack protections, though the tables suggest this is true in a few cases. The more significant issue is that there is no effective mechanism for individuals to enforce their rights to an unharmed online personae. New legal protections, such as an extension to the EU Charter, would motivate all search engines and social media companies operating within the EU to modify their algorithms to deter online personae hacking.

The EU’s current reliance on risk-based compliance is supportive here (OECD, 2021). Penalties for failure to act and failure to implement regulations must be proportional and dissuasive of future abuses (Faure, 2010). That means that if an illegal action generates millions of Euros in profits, penalties must be significant enough to dissuade the firm from taking similar actions. Moreover, suppose the harm done is irreparable, like contributing significantly to an election’s outcome or recruitment for a terrorist attack. In that case, criminal sanctions against the firm and its highest officers may be appropriate, including prison time. Note that with risk-based compliance, the threshold for criminal action can be establishing a significant contribution to the event’s occurrence and does not require establishing sole responsibility for its occurrence. That should address issues of stochastic terrorism and stochastic reframing of the truth discussed above.

We are all familiar with the recent “right to be forgotten,” which does not remove something from the permanent and universal archive of the net but does remove it from the result lists returned by search algorithms. This can be seen as the equivalent of an “obligation to be discounted,” requiring the search engine to discount harmful and irrelevant or outdated information, protecting the individual invoking the “right to be forgotten” from further harm. It can also be seen as a new right, the “right not be deceived,” benefitting all users.

There are indeed two rights; thus, the platforms have two sets of obligations. The first obligation for a search engine or social media company is to provide users with the best available feeds matching their interests, subject to constraints on accuracy and timeliness. The second obligation for a search engine or platform is that items that fail a test on accuracy and truthfulness must not be displayed, even if they would delight the user and generate profits for a social media platform.

There need to be exceptions to these rules to enable researchers to work and to find all relevant historical documents. Still, these exceptions should not violate the rule that false and harmful disinformation should not be permitted to dominate the news feeds of any social media companies that operate within the relevant jurisdictions defined above. A search for “Ben Edelman” is no longer dominated by search results about his dispute with a Chinese restaurant, though these results can still be found.

### Redesign of search engine and social media algorithms

Ideally, users would recognize and reject disinformation campaigns and attempts to discredit a person by hacking their online presence. Unfortunately, there is ample evidence that this is not happening. Ideally, social media websites and curators of content would reject online personae hacking and carefully curate content, and search engines would not participate in persona hacking. Likewise, neither of these seems to be occurring. Moreover, the algorithmic creation of fakes of all kinds is making the problems of manipulated personae more severe. Perhaps the only solution is to use algorithmic detection and limit persona hacking. We explore this next.

Attempts to train users to recognize when false content has been posted, an essential element of hacking an online personae, have proven largely unsuccessful. User discretion cannot be advised, or at least it cannot be relied upon to detect hacking. The fake news community has explored labeling false and harmful content (A. Kim & Dennis, 2019; Kim et al., 2019a, 2019b), a form of debunking, with only limited success, and sites like Facebook have abandoned labeling (Pennycook & Rand, 2019; Pennycook et al., 2020). Other researchers have explored training users to detect and reject false and harmful content without requiring labeling, called pre-bunking, but that seems to offer limited promise as well (van der Linden et al., 2021): if most users are unwilling to pay attention to a warning label, how many would be willing to invest the time in a training program and then invest the effort needed to evaluate all content with or without labeling? Users will continue to be manipulated by false and harmful content, and individuals will suffer harm from persona hacking.

Likewise, it is unreasonable to expect the victim of a hacked online personae to detect every violation, initiate action to have the offending content removed, and then litigate if unable to obtain relief in any other fashion. Finally, as we have observed, for some forms of persona hacking, there are no legal remedies, and other remedies are far too slow or insufficient to rectify the harm that has been caused.

If users cannot be expected to detect false and harmful content, and if individuals cannot be expected to exercise perpetual vigilance to protect their online personae in the presence of persistent, sophisticated, well-executed disinformation campaigns, the only remaining alternative is to limit the posting of attacks on online personae. Fortunately, most online personae hacking does not involve a direct attack on the individuals’ or the organizations’ official sites. Most online personae hacking, like most disinformation campaigns, is performed through online platforms. These online platforms must now take an active role in limiting their roles in online abuse. The combination of a fundamental right in the EU and risk-based compliance should provide adequate motivation for websites to begin to take seriously their obligation to manage and moderate content on the website. And, while their users may lack the interest and the ability to detect false content, that cannot be true of websites. Regulation provides the necessary incentives if fines are sufficient to be dissuasive, as indeed they are required to be.

Legal scholars will debate the implications of the settlement between Dominion and Fox News for years to come, but there are two obvious conclusions. First, individuals like Sidney Powell, former President Trump’s attorney, can say whatever they want, protected by the First Amendment. Second, responsible news organizations and media companies cannot repeat the most absurd statements of these individuals as if they were true and cannot provide a platform for them to speak at length, without question, as if their comments were true. Libel no longer requires proof of malice; news organizations can be liable for libel if they knowingly propagate dangerous falsehoods or recklessly propagate falsehoods they should know to require verification. As Dominion’s attorney noted, “Lies have consequences” (Bauder, 2023) and lying to increase market share may become increasingly unprofitable.

Ultimately, a new fundamental right in the EU to an unharmed online personae, supported by risk-based compliance with that right, would affect the behavior of all platforms operating in the EU. Since the EU requires that penalties be dissuasive and proportional, a platform that earned hundreds of millions of Euros through enabling disinformation could expect comparable penalties. Note that this affects editorial and news content since the litigation between Dominion and Fox News was based solely on content aired by Fox’s opinion commentators (Levenson & Cohen, 2023) and not Fox’s news team members. Note also that this is separate from and in addition to existing restrictions limiting hate speech and calls for violence.

Any demand for an active policy limiting content posting must demonstrate that the policy is feasible and that algorithms to support it can indeed be implemented. The algorithm we propose is quite simple. Based on a combination of the source’s reliability, the emotional level and sensationalism of the wording, and the consistency of the message with other posted content, the message can either be approved for posting or denied approval. Something from a highly trusted source can be published even if it is not supported by content elsewhere on the web; indeed, that’s how investigative journalism works. Stories from an unreliable source, supported by a large number of unreliable posts, will not be approved for posting. The actual parameters will need to be adjusted until the right balance is achieved, and the algorithm may need to be tweaked as users learn to game the system. Algorithmically assessing the accuracy of online news sources is complicated. For one-off stories that go viral, there may be no historical basis for assessing accuracy unless it is possible to determine where the story actually originated.

There is a difference between bias, however, and outright falsehood and disinformation. There may be no genuinely unbiased websites, but an algorithm can be designed to explore a set of reliable sources and to adjust for their known biases. There are indeed lists and assessments of reliable sources, and guidelines are evolving for assessing the reliability of posts (The News Literacy Project, 2023). Some consensus probably already exists: *The New York Times* and *The Wall Street Journal* are reliable and factual, although their political orientations differ. NPR and Fox News have diametrically different political orientations, but the straight news coverage from both could probably be considered reliable. Posts originating at Russian, Iranian, or Chinese disinformation and troll factories can usually be identified as such (Nemr & Gangware, 2019). Citizens in totalitarian regimes may have reasons for concealing their identities before posting online, but this is rarely true in Western democracies. In the West, posts whose origin cannot be verified would be assumed to be of low reliability. Any content deemed unreliable would automatically be blocked by filtering algorithms at all major platforms.

This approach does not mean that content would be censored or suppressed. It simply means that the content could not be posted automatically. Our proposal would have blocked Daniel Ellsberg’s posting of the Pentagon Papers online as an individual. It would not have blocked *The New York Times* and *The Washington Post* from reviewing, verifying, and publishing them after verification. Likewise, our policy does not block content because it is harmful to an individual or an organization or damaging to their online personae; it blocks websites from automatically accepting a post that is damaging to an online personae if it is from an unreliable source.

Measures must be fair and objective and seen as fair and objective. Many individuals believe the truth of what they attempt to post or attempt to forward, and blocking their actions will naturally be seen as politically motivated. It may even be seen as an assault on free speech. Algorithms must be free of any bias, even though they will almost certainly be seen as biased by individuals whose attempted actions they limit.

## Conclusions, future research, and limitations

The dilemma we face today is that some forms of technology-enabled abuses of human dignity, specifically persona hacking, are not currently limited by laws and regulations. Moreover, attempts to protect personae from malevolent hacking may appear to contradict other rights, like freedom of speech. Before we can rectify omissions in the protection of online personae, we need to reassert our legal philosophy—that human dignity is a fundamental right—and that individuals, content creators, content distributors and platforms, and even foreign governments can be compelled to act in ways that protect human dignity. Human dignity is already acknowledged as a fundamental right. The problem is implementing and enforcing meaningful protections against persona hacking.

The classification we introduced in this discussion paper provides a way of systematically viewing abuses of online personae. They showed how and why existing laws and regulations do not yet cover individual forms of attack.

In Table 3, we describe the challenges associated with implementing protections against online personae hacking and show how each form of abuse requires different clarifications for regulators and legislators to implement meaningful protections.

**Table 3:** The nature of protections offered by a fundamental right to an unharmed persona

Name of technique	Description of problem	Nature of a new protection
Flooding the zone	A form of search engine “dis-optimization,” whether performed by the search engine or not, must be blocked. It is a new form of harm, a new form of misrepresentation, and not currently limited because it does not rely on false information	Since this occurs on the search engine, it needs to be prevented at the search engine. The solution does not seek to limit what any traditional media outlet can publish and does not interfere with freedom of speech. It prevents manipulation of the search engine to manipulate perceived persona. Search engines cannot engage in this activity, and they cannot permit manipulation of their results by others
Replacement and Google bombing	The courts in the US have established that a firm can buy another firm’s trademark as a search term. Is it just a small step towards hiding an individual's persona? If the persona is not being used by “the hider”—(if there is no impersonation)—is there any protection except trying to do a better job of search engine optimization to gain and retain attention? Can an individual ever “out-compete” the amusing collection of alternative funny stories?	What would a new right look like? Does the search engine have an obligation to disambiguate the way that Wikipedia chooses to disambiguate? Should a misleading link be suppressed? Should it be demoted or flagged in some way? This would require a new protection and would require broad cooperation across platforms
High-impact lying	This is clearly illegal defamation, but remediation is slow and, unless countered immediately, can result in irreparable harm, as in loss of elections	This runs squarely into the problem of disinformation and freedom of speech. It took a long time to force Fox News to stop its disinformation campaign against Dominion Voting. It still is not possible to stop Fox from lying about the election or individual officials. Section 230 protects websites. We need techniques to protect individuals from falsehood masquerading as free speech, and remediation is too slow and uncertain. What would a new protection look like? Who is entitled to protection? How do we deal with claims of censorship and violation of freedom of speech?
Creating stochastic truth and alternative facts	This involves waves of competing falsehoods, not designed to convince a population of an alternative “truth” but to confuse the population so totally that the search for truth is abandoned. Putin’s Russia uses this extensively at home to demoralize the people into believing that the truth is unknowable, there are no virtuous players, and the current policy cannot be as bad as it may initially appear	This is especially difficult to counter. False stories can be provided, especially by foreign troll factories, taken down, and replaced with new stories as quickly as the old ones are removed. A constantly changing truth is consistent with the objectives of this form of persona hacking. Moreover, we must determine when these stories are parodies and protected social commentary. When are they lies provided by a foreign power? What defense is possible when the intent is not to deceive but merely to confuse? How can any form of liability be established when the harm from any single story is unclear (stochastic harm)?

We do not underestimate the magnitude of the challenge we place in front of regulators. When we look at our classification of threats, we see that each falls outside of the forms of relief currently available to individuals to protect them from reputational harm and those available to brands to protect them from loss of brand equity and reputational damage. It is not immediately clear how the EU or US, or any of their national legislative bodies, should respond to create simple, consistent, and enforceable relief or even how to draft legislation that does not violate current protections on free speech. Moreover, any new policy would require an active role for platforms in their implementation, something that platform operators have been unwilling to contemplate. Table 3 describes the difficulties in creating a regulatory response to each form of persona hacking. Each will require a policy that can be accepted, that is, does not violate currently held beliefs concerning free speech and censorship. Each will require some degree of cooperation from platform operators, coerced if necessary, and with penalties that increase exponentially over time. Since persona hacking during an election is time-critical, the ability to coerce appropriate behavior must include penalties for inaction that become draconian quite quickly. Audio–video media-rich persona hacking is evolving so rapidly that we need to defer the development of an analysis corresponding to Table 3.

Legal scholars will note that we have ignored intent, that is, the presence or absence of malice, as a criterion for requiring platforms to act. The stories used to manipulate an online personae may actually be true. The platform operator’s algorithm may determine that certain stories deserve high placement in their search results because of skillful, successful, and malicious search engine manipulation by parties hostile to the person whose persona is being hacked. But neither the absence of falsehood nor malice would serve as sufficient justification for inaction by the search engine operator. This is consistent with a recent decision of the European Court of Justice involving the “right to be forgotten” in Google v. Spain ("Case C-131/12, Google Spain and Google," 2014), but goes further. It may indeed be a step too far for some scholars.

We have no feasibility study to address if our recommendations are acceptable to the community of legal scholars in the EU or the US or if our recommendations could lead to policies that can be clearly articulated. We do not know if policies could be implemented in either jurisdiction, given the power of platform operators, the power of their lobbying efforts to hinder restrictions on their actions, and their demonstrated history of circumventing restrictions. And we also do not know if algorithms to limit the spread of harmful content on social media would work as intended. However, we hope to start this critical discussion in the community of information systems and legal scholars and beyond.

## References

[CR1] Alavi, S. (2018). Online defamation law: The future requires more. *The Advocates’ Quarterly,**49*, 133.

[CR2] Albahar, M., & Almalki, J. (2019). Deepfakes: Threats and countermeasures systematic review. *Journal of Theoretical and Applied Information Technology,**97*(22), 3242–3250.

[CR3] Ausloos, P. R. J. (2020). *The right to erasure in EU data protection law*. Oxford University Press.

[CR4] Baghramian, M. (2020). *From trust to trustworthiness*. Routledge.

[CR5] Bankoff, C. (2014, December 9). *Harvard Business School professor fails to bully Chinese restaurant into giving him $12*. New York Magazine. https://nymag.com/intelligencer/2014/12/hbs-professor-fails-to-bully-restaurant.html

[CR6] Barbour, K., Lee, K., & Moore, C. (2017). online personae research: An Instagram case study. *Persona Studies,**3*(2), 1–12. 10.3316/informit.955808284201687

[CR7] Bauder, D., Chase, R., & Mulvihill, G. (2023, April 18). *Fox News pays Dominion Voting Systems $787.5 million to settle defamation suit over election lies with trial imminent*. Fortune. https://fortune.com/2023/04/18/fox-news-settles-dominion-voting-systems-defamation-lawsuit-jury-seated/

[CR8] Bell, B. T. (2019). "You take fifty photos, delete forty nine and use one”: A qualitative study of adolescent image-sharing practices on social media. *International Journal of Child-Computer Interaction,**20*, 64–71. https://doi.org/10.1016/j.ijcci.2019.03.002

[CR9] Bergo, B. (2019). Emmanuel Levinas. In E. N. Zalta (Ed.), *The Stanford Encyclopedia of Philosophy* (Fall 2019 ed.). https://plato.stanford.edu/archives/fall2019/entries/levinas/

[CR10] Berman, T. (2014). *Miserly Harvard professor harasses Chinese restaurant over $4*. Gawker. Retrieved September 20, 2022 from https://www.gawker.com/miserly-harvard-professor-harasses-chinese-restaurant-o-1668937632

[CR11] Bond, S. (2021, May 14). *Just 12 people are behind most vaccine hoaxes on social media, Research Shows*. NPR. https://www.npr.org/2021/05/13/996570855/disinformation-dozen-test-facebooks-twitters-ability-to-curb-vaccine-hoaxes

[CR12] Bradt, S. (2020, January 10). *MIT releases results of fact-finding on engagements with Jeffrey Epstein*. MIT News. https://news.mit.edu/2020/mit-releases-results-fact-finding-report-jeffrey-epstein-0110

[CR13] Brady. (2004, November 13). *Google, Microsoft no longer ‘more evil than Satan’*. Microsoft Bing Blog. https://blogs.bing.com/search/2004/11/13/google-microsoft-no-longer-more-evil-than-satan

[CR14] Bryne, J. A. (2023, February 17). *The Harvard Business School prof who acted like a jerk & lost tenure is now suing HBS. Here’s why he should lose*. Poets & Quants. https://poetsandquants.com/2023/02/17/the-harvard-business-school-prof-who-acted-like-a-jerk-lost-tenure-is-now-suing-hbs-heres-why-he-should-lose/

[CR15] Bullingham, L., & Vasconcelos, A. C. (2013). The presentation of self in the online world’: Goffman and the study of online identities. *Journal of Information Science,**39*(1), 101–112. 10.1177/0165551512470051

[CR16] Burns, H. (2023, February 15). *Did a viral spat over Chinese restaurant prices cost Harvard professor tenure?* The Boston Globe. https://www.boston.com/news/the-boston-globe/2023/02/15/harvard-business-school-professor-benjamin-edelman-tenure-chinese-restaurant/

[CR17] Casey, E. S. (2006). The ethics of the face-to-face encounter: Schroeder, Levinas, and the Glance. *The Pluralist,**1*(1), 74–97. https://www.jstor.org/stable/20708851

[CR18] Chekhov, A. (2000). *Selected stories of Anton Chekhov*. Penguin Random House.

[CR19] Chesney, B., & Citron, D. (2019). Deep fakes: A looming challenge for privacy, democracy, and national security. *California Law Review,**107*, 1753–1820. 10.15779/Z38RV0D15J

[CR20] Clarke, R. (1994). The digital persona and its application to data surveillance. *The Information Society,**10*(2), 77–92. 10.1080/01972243.1994.9960160

[CR21] Clarke, R. (2014). Persona missing, feared drowned: The digital persona concept, two decades later. *Information Technology and People,**27*(2), 182–207. 10.1108/ITP-04-2013-0073

[CR22] Claypoole, T., & Payton, T. (2016). *Protecting your internet identity: Are you naked online?* Rowman & Littlefield.

[CR23] Darcy, O., & Maghe, S. (2022, November 11).* Alex Jones ordered to pay nearly half a billion dollars to Sandy Hook families in additional damages*. CNN. https://edition.cnn.com/2022/11/10/media/alex-jones-sandy-hook-damages/index.html

[CR24] De Kerckhove, D., & Miranda De Almeida, C. (2013). What is a digital persona? *Technoetic Arts: A Journal of Speculative Research,**11*(3), 277–287. 10.1386/tear.11.3.277_1

[CR25] Dossa, S. (1989). *The public realm and the public self: The political theory of Hannah Arendt*. Wilfried Laurier University Press.

[CR26] Farchy, J. (2024, January 24). *Oil trader sues UAE claiming smear campaign bankrupted his firm*. https://www.bloomberg.com/news/articles/2024-01-24/oil-trader-sues-uae-claiming-smear-campaign-bankrupted-his-firm

[CR27] Faure, M. G. (2010). Effective, proportional and dissuasive penalties in the implementation of the environmental crime and shipsource pollution directives: Questions and challenges. *European Energy and Environmental Law Review,**19*(6), 256–278. 10.54648/eelr2010019

[CR28] Fawkes, J. (2015). Performance and persona: Goffman and Jung’s approaches to professional identity applied to public relations. *Public Relations Review,**41*(5), 675–680. 10.1016/j.pubrev.2014.02.011

[CR29] Finger, L. (2022, September 8). *Overview of how to create deepfakes – It’s scarily simple*. Forbes. https://www.forbes.com/sites/lutzfinger/2022/09/08/overview-of-how-to-create-deepfakesits-scarily-simple/?sh=90f53022bf16

[CR30] Flitter, E., & Stewart, J. B. (2021, October 18). *Bill Gates met with Jeffrey Epstein Many times, despite his past*. The New York Times. https://www.nytimes.com/2019/10/12/business/jeffrey-epstein-bill-gates.html

[CR31] Fox, J. C. (2021, March 26). Harvard sanctions professor with close Jeffrey Epstein ties, closes program he ran. *Boston*. https://www.boston.com/news/local-news/2021/03/26/harvard-sanctions-professor-with-close-jeffrey-epstein-ties-closes-program-he-ran/

[CR32] Gerstein, J., & Ward, A. (2022, May 3). What falls after Roe? Liberals warn of a privacy rights nightmare. *Politico*. https://www.politico.com/news/2022/05/03/supreme-court-abortion-privacy-rights-00029871

[CR33] Gilbert, B. (2019, September 10). *An elite group within one of America’s most prestigious universities is embroiled in the ongoing Jeffrey Epstein scandal, and its director just quit — Here’s what’s going on*. Insider. https://www.businessinsider.com/mit-media-lab-connection-to-jeffrey-epstein-explained-2019-9

[CR34] Gillespie, B., & Cina, M. (2023, March 16). *Biden Blunders: President stumbles his way through gaffe-filled winter*. Fox News. https://www.foxnews.com/politics/joe-biden-blunders-president-stumbles-through-gaffe-filled-winter

[CR35] Goffman, E. (1956). *The presentation of self in everyday life*. University of Edinburgh Social Sciences Research Centre.

[CR36] Gold, M., & Ashford, G. (2023, December 1). *George Santos is kicked out of congress in a historic vote*. The New York Times. https://www.nytimes.com/2023/12/01/nyregion/santos-expulsion-vote-congress.html#:~:text=George%20Santos%2C%20the%20New%20York,bipartisan%20vote%20by%20his%20peers

[CR37] Goldberg, M. (2016, July 24). *The Hillary haters*. Slate Magazine. https://www.slate.com/articles/news_and_politics/cover_story/2016/07/the_people_who_hate_hillary_clinton_the_most.html

[CR38] Google Spain SL and Google Inc. v Agencia Española de Protección de Datos (AEPD) and Mario Costeja González (2014). Court of Justice of the European Union, Case C‑131/12.

[CR39] Google. (2022). *Reasons for removal of content from Google provided by government or court entities in the United States during 2nd half of 2022*. Statista. Retrieved November 24, 2023, from https://www.statista.com/statistics/1128937/us-government-reasons-requests-for-content-removal-google/

[CR40] Google. (2023). *Reasons for government and court requests for content removal from Google India from January to June 2023*. Statista. Retrieved November 24, 2023, from https://www.statista.com/statistics/1219091/india-reasons-for-content-removal-requests-from-government/

[CR41] Greenberg, Z. (2019, July 11). *How Jeffrey Epstein made himself into a ‘Harvard man’*. Boston Globe. https://www.bostonglobe.com/metro/2019/07/11/how-jeffrey-epstein-made-himself-into-harvard-man/m672RjwFJFwWOVzF9WRNjO/story.html

[CR42] Hamilton, P. A. (2013). *Google-bombing - Manipulating the PageRank algorithm*. Information Retrieval*. *https://api.semanticscholar.org/CorpusID:10760843

[CR43] Hancock, J. T., & Bailenson, J. N. (2021). The social impact of deepfakes. *Cyberpsychology, Behavior, and Social Networking,**24*(3), 149–152. 10.1089/cyber.2021.29208.jth 33760669 10.1089/cyber.2021.29208.jth

[CR44] Harris, D. (2019). Deepfakes: False pornography is here and the law cannot protect you. *Duke Law & Technology Review,**17*(1), 99–127.

[CR45] Harwell, D. (2018, November 8). *White House shares doctored video to support punishment of journalist Jim Acosta*. The Washington Post. https://www.washingtonpost.com/technology/2018/11/08/white-house-shares-doctored-video-support-punishment-journalist-jim-acosta/

[CR46] Henderson, P., Hashimoto, T., & Lemley, M. (2023). Where’s the liability in harmful AI speech? *Journal of Free Speech Law,**3*, 589–650. 10.48550/arXiv.2308.04635

[CR47] Heugas, A. C. (2021). Protecting image rights in the face of digitalization: A United States and European analysis. *The Journal of World Intellectual Property,**24*(5–6), 344–367. 10.1111/jwip.12194

[CR48] James, W. (1890). *The Principles of Psychology*. Holt.

[CR49] John, A. (2020, June 23). *From birtherism to ‘treason’: Trump’s false allegations against Obama*. Los Angeles Times. https://www.latimes.com/politics/story/2020-06-23/trump-obamagate-birtherism-false-allegations

[CR50] Jung, C. G. (1953). *Two essays on analytical psychology*. Routledge.

[CR51] Kamenetz, A. (2017, October 31). *Learning to spot fake news: Start with a gut check*. NPR. https://www.npr.org/sections/ed/2017/10/31/559571970/learning-to-spot-fake-news-start-with-a-gut-check

[CR52] Katz, R., Ogilvie, S., Shaw, J., & Woodhead, L. (2022). *Gen Z, explained: The art of living in a digital age*. University of Chicago Press.

[CR53] Kietzmann, J., Lee, L. W., McCarthy, I. P., & Kietzmann, T. C. (2020). Deepfakes: Trick or treat? *Business Horizons,**63*(2), 135–146. 10.1016/j.bushor.2019.11.006

[CR54] Kim, A., & Dennis, A. R. (2019). Says who? The effects of presentation format and source rating on fake news in social media. *MIS Quarterly,**43*(3), 1025–1039. 10.25300/MISQ/2019/15188

[CR55] Kim, A., Moravec, P. L., & Dennis, A. R. (2019a). Combating fake news on social media with source ratings: The effects of user and expert reputation ratings. *Journal of Management Information Systems,**36*(3), 931–968. 10.1080/07421222.2019.1628921

[CR56] Kim, H., Elgharib, M., Zollhöfer, M., Seidel, H. P., Beeler, T., Richardt, C., & Theobalt, C. (2019b). Neural style-preserving visual dubbing. *ACM Transactions on Graphics,**38*(6), 1–13. 10.1145/3355089.3356500

[CR57] Kirkpatrick, D. D. (2023, MArch 27). *The dirty secrets of a smear campaign*. The New Yorker. https://www.newyorker.com/magazine/2023/04/03/the-dirty-secrets-of-a-smear-campaign

[CR58] Klepper, D. (2023, January 24). *Learning to lie: AI tools adept at creating misinformation*. APNews. https://apnews.com/article/technology-science-business-artificial-intelligence-afb4618ff593db9e3e51ecbd91dc3eef

[CR59] Lambert, P. (2019). *The right to be forgotten: Interpretation and practice*. Bloomsbury Professional.

[CR60] Lamphere, R. A., & Leary, M. R. (1990). Private and public self-processes: A return to James’s constituents of the self. *Personality and Social Psychology Bulletin,**16*(4), 717–725. 10.1177/0146167290164012

[CR61] Langa, J. (2021). Deepfakes, real consequences: Crafting legislation to combat threats posed by deepfakes. *Boston University Law Review,**101*, 761–801.

[CR62] Levenson, E., & Cohen, M. (2023, April 17). *Here are the 20 specific Fox broadcasts and tweets Dominion says were defamatory*. CNN Business. https://www.cnn.com/2023/04/17/media/dominion-fox-news-allegations/index.html

[CR63] Levinas, E. (1961). *Totalité et Infini: Essai sur l’extériorité*. Martinus Nijhoff.

[CR64] Mansfield-Devine, S. (2023). Weaponising ChatGPT. *Network Security*, *2023*(4). 10.12968/S1353-4858(23)70017-2

[CR65] Martínez, J. M., & Mecinas, J. M. (2018). Old wine in a new bottle? Right of publicity and right to be forgotten in the internet era. *Journal of Information Policy,**8*(1), 362–380. 10.5325/jinfopoli.8.2018.0362

[CR66] Mead, G. H. (1982). *The individual and the social self: Unpublished essays by G. H. Mead* (D. L. Miller, Ed.). University of Chicago Press.

[CR67] Metz, C. (2023, May 1). *The godfather of A.I. leaves Google and warns of danger ahead*. The New York Times. https://www.nytimes.com/2023/05/01/technology/ai-google-chatbot-engineer-quits-hinton.html

[CR68] Miller, J. R. (2022, March 17). *Deepfake video of Zelensky telling Ukrainians to surrender removed from social platforms.* New York Post. https://nypost.com/2022/03/17/deepfake-video-shows-volodymyr-zelensky-telling-ukrainians-to-surrender/

[CR69] Moore, C., Barbour, K., & Lee, K. (2017). Five dimensions of online persona. *Persona Studies,**3*(1), 1–11. 10.3316/informit.956460438195724

[CR70] Nemr, C., & Gangware, W. (2019). *Weapons of mass distraction: Foreign state-sponsored disinformation in the digital age*. Park Advisors. https://www.state.gov/wp-content/uploads/2019/05/Weapons-of-Mass-Distraction-Foreign-State-Sponsored-Disinformation-in-the-Digital-Age.pdf

[CR71] O’Connell, A. (2020). Image rights and image wrongs: Image-based sexual abuse and online takedown. *Journal of Intellectual Property Law & Practice,**15*(1), 55–65. 10.1093/jiplp/jpz150

[CR72] OECD. (2021). Risk-based regulation OECD regulatory policy outlook 2021. *OECD Publishing*. 10.1787/38b0fdb1-en

[CR73] Owens, J. (1988). The self in Aristotle. *The Review of Metaphysics,**41*(4), 707–722. https://www.jstor.org/stable/20128658

[CR74] Pennycook, G., & Rand, D. G. (2019). Fighting misinformation on social media using crowdsourced judgments of news source quality. *Proceedings of the National Academy of Sciences,**116*(7), 2521–2526. 10.1073/pnas.1806781116 10.1073/pnas.1806781116 PMC6377495 30692252

[CR75] Pennycook, G., Bear, A., Collins, E. T., & Rand, D. G. (2020). The implied truth effect: Attaching warnings to a subset of fake news headlines increases perceived accuracy of headlines without warnings. *Management Science,**66*(11), 4944–4957. 10.1287/mnsc.2019.3478

[CR76] Plenta, P. (2020). Conspiracy theories as a political instrument: Utilization of anti-Soros narratives in Central Europe. *Contemporary Politics,**26*(5), 512–530. 10.1080/13569775.2020.1781332

[CR77] Posters, B. (2019, June 13). *‘I wish I could....’*. Instagram. https://www.instagram.com/bill_posters_uk/p/BypkGIvFfGZ/

[CR78] Raffel, S. (2013). The everyday life of the self: Reworking early Goffman. *Journal of Classical Sociology,**13*(1), 163–178. 10.1177/1468795X12474055

[CR79] Reuters. (2020, October 29). *Fact check: Clip of Biden taken out of context to portray him as plotting a voter fraud scheme*. https://www.reuters.com/article/uk-fact-check-biden-voter-protection-not-idUSKBN27E2VH

[CR80] Revilla, C. (2017). *As social media continues to evolve, online defamation laws remain stagnant*. FIU Law Review. https://law.fiu.edu/2017/04/17/social-media-continues-evolve-online-defamation-laws-remain-stagnant/

[CR81] Roozenbeek, J., & Linden, S. (2019). Fake news game confers psychological resistance against online misinformation. *Palgrave Communications*,* 5*(65). 10.1057/s41599-019-0279-9

[CR82] Sadagheyani, H. E., & Tatari, F. (2021). Investigating the role of social media on mental health. *Mental Health and Social Inclusion,**25*(1), 41–51. 10.1108/MHSI-06-2020-0039

[CR83] Shulman, D. (2022). Self-presentation: Impression management in the digital age. In M. H. Jacobsen & G. Smith (Eds.), *The Routledge International Handbook of Goffman Studies* (pp. 26–37). Routledge.

[CR84] Spivak, R. (2019). “Deepfakes”: The newest way to commit one of the oldest crimes. *Georgetown Law Technology Review,**3*(2), 339–400.

[CR85] Stalin, J. S. D., & Roy, D. D. (2019, April 19). *Elections 2019: Did Google CEO Sundar Pichai vote? Viral Photo Fact Checked*. https://www.ndtv.com/india-news/lok-sabha-polls-2019-did-google-ceo-sundar-pichai-vote-today-viral-photo-fact-checked-2025098

[CR86] Stern, M. J. (2014, November 20). Speaking in code. *Slate Magazine*. https://slate.com/technology/2014/11/are-google-results-free-speech-protected-by-the-first-amendment.html

[CR87] Stolz, S. (2020). Nietzsche’s psychology of the self: The art of overcoming the divided self. *Human Arenas,**3*, 264–278. 10.1007/s42087-019-00081-x

[CR88] Susser, D., Roessler, B., & Nissenbaum, H. (2019). Online manipulation: Hidden influences in a digital world. *Georgetown Law Technology Review,**4*(1), 1–52.

[CR89] The News Literacy Project. (2023). Retrieved November 12, 2023, from https://newslit.org/for-everyone/

[CR90] TNM. (2017, June 2). *Sundar Pichai is the new bad meme:* ‘*Quotes*’ *that the Google CEO wouldn’t have dreamed of making*. https://www.thenewsminute.com/social/sundar-pichai-new-bad-meme-quotes-google-ceo-wouldnt-have-dreamed-making-63046

[CR91] U.S. Attorney's Office. (2023, May 10). *Congressman George Santos charged with fraud, money laundering, theft of public funds, and false statements*. Retrieved November 12, 2023, from https://www.justice.gov/usao-edny/pr/congressman-george-santos-charged-fraud-money-laundering-theft-public-funds-and-false

[CR92] UK House of Commons. (2018). *Disinformation and ‘fake news’*. Retrieved November 14, 2023, from https://publications.parliament.uk/pa/cm201719/cmselect/cmcumeds/1791/179102.htm

[CR93] van der Linden, S., Dixon, G., Clarke, C., & Cook, J. (2021). Inoculating against COVID-19 vaccine misinformation. *eClinicalMedicine*, 33, 100772. 10.1016/j.eclinm.2021.100772 10.1016/j.eclinm.2021.100772 PMC7908879 33655205

[CR94] van Huijstee, M., van Boheemen, P., Das, D., Nierling, L., Jahnel, J., Karaboga, M., & Fatun, M., Martin. (2021). *Tackling deepfakes in European policy*. European Parliament Research Service. https://www.europarl.europa.eu/RegData/etudes/STUD/2021/690039/EPRS_STU(2021)690039_EN.pdf

[CR95] Vertuno, J. (2022, August 5). *Alex Jones ordered to pay $49.3M in total damages over false Sandy Hook conspiracy theories*. PBS. https://www.pbs.org/newshour/nation/alex-jones-ordered-to-pay-49-3m-in-total-damages-over-false-sandy-hook-conspiracy-theories

[CR96] Vogelstein, F. (2018, July 25). *Facebook just learned the true cost of fixing its problems*. Wired. https://www.wired.com/story/facebook-just-learned-the-true-cost-of-fixing-its-problems/

[CR97] Washington Post. (2019a, June 25). *How to spot manipulated video | The Fact Checker*. YouTube. https://www.youtube.com/watch?v=RVrANMAO7Sc

[CR98] Washington Post. (2019b, May 23). *Pelosi videos manipulated to make her appear drunk are being shared on social media*. YouTube. https://www.youtube.com/watch?v=sDOo5nDJwgA

[CR99] Weil, E. (2023, March 1). *You are not a parrot*. Intelligencer - NY Magazine. https://nymag.com/intelligencer/article/ai-artificial-intelligence-chatbots-emily-m-bender.html

[CR100] Werro, F. (2020). *The right to be forgotten: A comparative study of the emergent right’s evolution and application in Europe, the Americas, and Asia*. Springer Nature. 10.1007/978-3-030-33512-0

[CR101] Westerlund, M. (2019). The emergence of deepfake technology: A review. *Technology Innovation Management Review,**9*(11), 39–52.

[CR102] Wikipedia. (2023a). *Google bombing*. Retrieved November 14, 2023, from https://en.wikipedia.org/wiki/Google_bombing

[CR103] Wikipedia. (2023b). *Political Google bombs in the 2004 U.S. presidential election*. Retrieved November 12, 2023, from https://en.wikipedia.org/wiki/Google_bombing

[CR104] Wikipedia. (2023c). *Sandy Hook Elementary School shooting conspiracy theories*. Retrieved November 12, 2023, from https://en.wikipedia.org/wiki/Sandy_Hook_Elementary_School_shooting_conspiracy_theories

[CR105] Williamson, E. (2021, November 15). *Alex Jones loses by default in remaining Sandy Hook defamation suits*. The New York Times. https://www.nytimes.com/2021/11/15/us/politics/alex-jones-sandy-hook.html

[CR106] Woodcock, A. (2020, June 29). *Online ‘pandemic of misinformation’ poses existential threat to UK’s democracy, report says*. The Independent. https://www.independent.co.uk/news/uk/politics/government-ministers-digital-age-report-uk-democracy-a9590121.html

